# Mechanisms of Strain Diversity of Disease-Associated in-Register Parallel β-Sheet Amyloids and Implications About Prion Strains

**DOI:** 10.3390/v11020110

**Published:** 2019-01-28

**Authors:** Yuzuru Taguchi, Hiroki Otaki, Noriyuki Nishida

**Affiliations:** 1 Division of Cellular and Molecular Biology, Nagasaki University Graduate School of Biomedical Sciences, Nagasaki 852-8523, Japan; noribaci@nagasaki-u.ac.jp; 2 Center for Bioinformatics and Molecular Medicine, Nagasaki University Graduate School of Biomedical Sciences, Nagasaki 852-8523, Japan; otaki@nagasaki-u.ac.jp

**Keywords:** α-synuclein, tau, amyloid, prion, prion protein, strain diversity, molecular dynamics simulation, secondary structure prediction, β-arch, in-register parallel β-sheet

## Abstract

The mechanism of prion strain diversity remains unsolved. Investigation of inheritance and diversification of protein-based pathogenic information demands the identification of the detailed structures of abnormal isoforms of the prion protein (PrP^Sc^); however, achieving purification is difficult without affecting infectivity. Similar prion-like properties are recognized also in other disease-associated in-register parallel β-sheet amyloids including Tau and α-synuclein (αSyn) amyloids. Investigations into structures of those amyloids via solid-state nuclear magnetic resonance spectroscopy and cryo-electron microscopy recently made remarkable advances due to their relatively small sizes and lack of post-translational modifications. Herein, we review advances regarding pathogenic amyloids, particularly Tau and αSyn, and discuss implications about strain diversity mechanisms of prion/PrP^Sc^ from the perspective that PrP^Sc^ is an in-register parallel β-sheet amyloid. Additionally, we present our recent data of molecular dynamics simulations of αSyn amyloid, which suggest significance of compatibility between β-sheet propensities of the substrate and local structures of the template for stability of amyloid structures. Detailed structures of αSyn and Tau amyloids are excellent models of pathogenic amyloids, including PrP^Sc^, to elucidate strain diversity and pathogenic mechanisms.

## 1. Introduction

Strain diversity is one of the most mysterious features of mammalian prions. The strain-specific traits of prions are enciphered in the structures of the abnormal isoform prion protein (PrP^Sc^), and they are stably inherited over generations through template-directed refolding of the normal isoform prion protein (PrP^C^), where the template PrP^Sc^ imprints the structural details onto the substrate PrP^C^ [1]. Moreover, the strain-specific pathogenic information encoded in the conformation of PrP^Sc^ is reproducibly “translated” into the strain-specific clinicopathological traits in manifested diseases [2,3]. This view is widely accepted as the protein-only hypothesis but detailed mechanisms, e.g., specifically what structures of PrP^Sc^ encode pathogenic information and how the information is translated, are unknown. Investigations of the storage, inheritance, and diversification of the protein-based pathogenic information warrants identification of structure–phenotype correlations; however, such a feat is difficult because detailed structures of the entire PrP^Sc^ is not available yet due to its incompatibility with high-resolution structural analyses, i.e., X-ray crystallography or nuclear magnetic resonance spectrometry (NMR). The incompatibility is attributable to difficulty in purifying without losing infectivity [4], in recapitulating bona fide PrP^Sc^ prions in-vitro with infectivity and toxicity, and relatively large sizes of PrP with post-translational modifications. Solid-state NMR (ssNMR) revealed in-register parallel β-sheet structures of in vitro-formed amyloids of the peptide corresponding to residues 23–144 [5], and ssNMR and electron paramagnetic resonance analyses have also indicated parallel in-register architectures for in vitro-formed amyloids of PrP residues 90–231 [6,7,8], but the structures of the whole molecule would be necessary for elucidation of strain diversity. Based on such data and other empirical constraints for PrP^Sc^, Groveman et al. propounded in-register parallel β-sheet structure models of PrP^Sc^ encompassing residues 90–231 [8]. However, the validity of these models for PrP^Sc^ is yet to be determined. On the other hand, cryo-electron microscopy (cryo-EM) of PrP^Sc^ lead to the four-rung β-solenoid model [9], although the resolutions were not high enough for atomic-level modeling. Which model is more plausible is still controversial in the field. As for the translation of the strain-specific structures, interactions of PrP^Sc^ with environments and/or other proteins are essential because each strain recognizes preferable cell groups to manifest the strain-specific lesion profiles, which provide the environment and factors required for efficient propagation [10,11,12].

**Figure 1:**
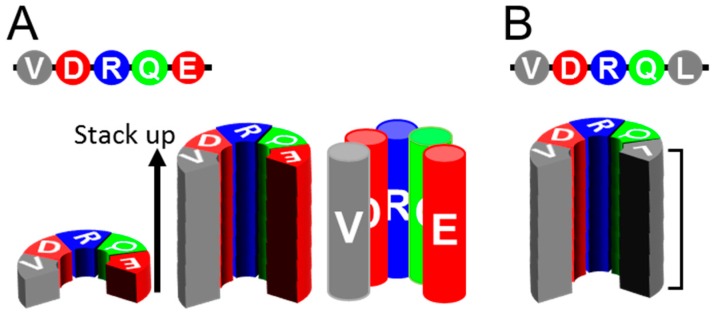
Uniqueness of in-register parallel β-sheet structures. (**A**). Schematics illustrating the primary structure of a peptide (top), a layer of the peptide in β-sheet structure (lower left), a stack of the in-register parallel β-sheets of the peptide (middle). Note that the identical groups of amino acids align along the fibril axis of the amyloid with ~4.8 Å intervals, like columns of the same groups of amino acids (right). (**B**). An example of a substitution mutation of the peptide, Leu for Glu (top). The substitution replaces a whole column (bracket) from the negatively-charged to the hydrophobic, creating a large hydrophobic patch encompassing the entire length of the fibril.

Many clinically important neurodegenerative diseases are associated with amyloids, e.g., Alzheimer’s disease (AD) with β-amyloid and Tau amyloid; Parkinson’s disease (PD) and dementia with Lewy bodies (DLB) with α-synuclein amyloids; and tauopathies, including Pick’s disease, with Tau amyloids. Interestingly, they show “prion-like properties,” including transmissibility to animals and even strain diversity [13,14,15]. Although they have no amino acid homology, those amyloids share a common basic architecture, in-register parallel β-sheet structure [14,16,17], which is characterized by a stack of β-loop-β motifs with identical groups of amino acids aligned along the fibril axis with narrow 4.8 Å intervals (Figure 1A). A given residue of the amyloid therefore can interact most frequently with the identical group counterparts of the adjacent layers. This structural feature may accentuate the characteristics of each residue of the peptide and greatly contribute to amyloid conformations, e.g., hydrophobic residues make extensive hydrophobic patches spanning along the entire fibril axis, while charged residues may disorder the local structures by repulsion between the layers and be preferentially exposed to the solvent. This feature also explains why a single mutation greatly affects properties and structures of the amyloid because it replaces the entire column (Figure 1B). Why do different proteins take the same in-register parallel β-sheet structures that can efficiently propagate? The in-register amyloids are hypothesized to have lower free energy than native conformations [18]. The hydrogen bonds between the backbones of the amyloid thermodynamically favor the in-register alignment [19]. Roterman et al. regarded the in-register amyloid as a “ribbon-like micelle” in which exposed hydrophobicity at the stacked ends enable endless elongation [20]; this would hold true even in the four-rung β-solenoid model of PrP^Sc^. Generally proteins with hydrophobic β-sheets exposed on the molecular surface are prone to aggregation formation. Soluble physiologically β-sheet-rich proteins have structural features to protect the solvent-facing β-sheets from undesired interactions and aggregation with other molecules [21]. Lack of such built-in protection systems in misfolded proteins, either in-register parallel β-sheets or β-solenoids, would allow uninhibited propagation of amyloids. Efficient propagation of the amyloids may stem from those thermodynamic features of the in-register parallel β-sheet structures. In-register parallel β-sheet amyloids are suitable experimental objects for molecular dynamics (MD) simulation if the structures of the amyloid are autonomously determined depending on the primary structure and thermodynamic principles.

Recent progresses in the structural analyses of disease-associated amyloids are even greater than those of PrP^Sc^ prions, partly because of their relatively small size, lack of post-translational modification, and in-vitro reproducibility of the cytotoxicity and infectivity of the amyloids [22]. Resolutions of the structures of those in-register amyloids determined by ssNMR or cryo-EM [14,17,23,24,25] are high enough to allow analyses of structure–phenotype relations. Here, we review recent advances in investigations of the structure and strain diversity of disease-associated in-register parallel β-sheet amyloids. Moreover, we herein introduce our in-silico data on the αSyn amyloid and discuss whether obtained knowledge is applicable to PrP^Sc^. To note, we particularly focus on relatively large pathogenic amyloids, such as α-synuclein and Tau.

## 2. Progress in Investigation of Tau Amyloids

The Tau protein is abundantly expressed in the nervous system, especially in nerve cell axons, and intrinsically disordered with a low content of secondary structures [26]. Tau has six isoforms depending on patterns of alternative splicing of exons 2, 3, and 10. Exon 10 corresponds to the second repeat of the four-repeat motif of the C-terminal microtubule-binding domain, and the absence or presence of exon 10 results in “three-repeat” (3R) or “four-repeat” (4R) Tau, respectively. The repeat motifs convert to an in-register parallel β-sheet structure common in tauopathies. Interestingly, which of the six isoforms preferentially converts to amyloids varies depending on disease types. For example, in Pick’s disease, the amyloids consist predominantly of the 3R-Tau, whereas AD amyloids contain both 3R- and 4R-Tau [27,28].

Recent work advanced our knowledge of the mechanism of strain diversity of amyloids, wherein cryo-EM detailed structures of two distinct Tau amyloids isolated from the brains of AD and Pick’s disease patients [14,25]. The distinct structures of the Tau amyloids demonstrated unequivocal examples of strain-specific structures. One of the notable differences between AD Tau and Pick’s Tau amyloids is the position of acute-angle β-arches, which was reminiscent of the mechanism of strain diversity of a yeast prion Sup35 postulated by Kajava et al. [29]. Interestingly, except for one β-sheet that is straight in the Pick’s fold but bent in the AD fold to make a new β-arch, many of the β-sheet regions in the AD amyloid also maintained β-sheet structure in the Pick’s amyloid, as if the positions of β-sheets are fixed, albeit with different facing partners for cross-β spine formation. The primary structures might be significant determinants of positions of β-sheets in in-register parallel amyloids, and cross-β-spine formation may be necessary to stabilize β-sheets by concealing hydrophobic residues from water [30]. Consistent with different patterns of the cross-β spines, Pick’s form and the AD form of Tau amyloids showed different proteolytic-fragment patterns on immunoblots after trypsin digestion [31].

Falcon and colleagues suggested mechanisms of strain diversity of Tau amyloids, which is applicable to other amyloids as well. They postulated that steric conflicts of the branching Cβ of Val_300_ of 4R-Tau with the omega-like structure formed by Pro_270_-Gly-Gly-Gly_273_ of the 3R-Tau of Pick’s amyloid makes the 4R-Tau an incompatible substrate, hampering cross-seeding of 4R-Tau by the Pick’s amyloid [27,32,33]. This type of strain barrier, due to incompatibility of local structures between the substrate monomer and the template amyloid, is discussed in detail regarding αSyn and PrP^Sc^.

Shifts of cross-β spine pairs accompanied by alterations in the positions of β-arches enable generation of structural polymorphs, even by amyloids with relatively simple conformations like Tau amyloids. Therefore, the similar mechanisms may operate in the diversification of strains of other amyloids including PrP^Sc^. For example, this may possibly explain the mechanism of how smaller proteolytic fragments of 12 or 13 kDa of PrP^Sc^ are produced, depending on the disease types [34,35,36]. If the fragments are produced by cleavage at protease-sensitive sites of PrP^Sc^, their differential sizes imply a shift of these sites. Such a shift can occur via alterations of cross-β spine patterns and β-arches. For example, when conversion to β-sheets and formation of cross-β spines propagate to the whole molecule like a slider and zipper from an amyloid core/interface that is first converted by the template amyloid, it is conceivable that the position of the amyloid core/interface determines the pairing partners of the cross-β spines and that a process starting from another amyloid core results in a distinct pattern of cross-β spines. Indeed, in de novo synthesis of infectious PrP^Sc^ prions [8,37,38,39], infectious PrP^Sc^ can be induced by either type of in vitro-formed PrP fibril with a protease-resistant core in the N-terminal [40] or C-terminal region [41,42]. The Creutzfeldt-Jakob disease (CJD) with 13-kDa fragments, e.g., sporadic CJD with methionine homozygosity at the codon 129, showed shortened incubation periods in human-mouse chimeric PrP-expressing mice, corroborating the significance of structural variation in strain barriers of PrP^Sc^ prions [36,43].

## 3. Progress in Investigation of αSyn Amyloids

αSyn is a cytosolic protein abundantly expressed in neurons, representing ~1 % of the total cytoplasmic proteins [44]. Since αSyn was cloned as the “non-Aβ component” (NAC) from AD amyloids [45], the first-identified region encompassing residues 61–95 is often referred to as the NAC region. It is localized mainly in presynaptic nerve terminals and contributes to control of neurotransmitter releases [46]. In addition to the diversity of clinicopathological features among synucleinopathies, i.e., PD, DLB, and multiple system atrophy (MSA), familial PD shows clinical variations depending on the causative mutations in the SNCA gene, in age of onset, disease duration, and presence of pyramidal signs [47,48]. Variations in physicochemical properties of αSyn amyloids are also well-documented. For instance, in-vitro formed αSyn fibrils showed at least two types of morphologically-distinguishable strains, “ribbon” and “fibril” types, which are different in dimensions of the fibrils, blotting patterns of protease-resistant fragments, cytotoxicity, and optimal salt conditions for efficient in-vitro propagation [49,50]. Their secondary structures determined by ssNMR revealed strain-specific β-sheet distributions: the ribbon had stable β-sheet structures in the N-terminal region encompassing residues 1–38, whereas the corresponding region of the fibril type was disordered except for a short β-sheet 16–20. On the contrary, residues 44–57 were disordered in the ribbon, while they are in a β-sheet in the fibril type. Although the distal NAC region of the fibril type might be structurally varied [50], positions of β-sheets in the NAC regions were relatively similar between the ribbon- and the fibril-types [49,50,51], similar to Pick’s and AD’s Tau amyloids sharing many β-sheet regions. Besides in vitro-formed fibrils, αSyn amyloids derived from DLB- and MSA-affected brains also differed in the blotting patterns of proteolytic fragments [52]. Like the different optimal propagation conditions between the ribbon and the fibril, MSA-αSyn amyloid is formed in the milieu of oligodendrocytes, whereas DLB-αSyn amyloid is formed in the neurons [52]. Thus, αSyn amyloids can be a good model to investigate strain diversification of pathogenic amyloids because of unequivocal structural differences between strains and structure–phenotype correlations as suggested by proteolytic-fragment patterns.

Another vigorously attempted biochemical approach for structural information of αSyn amyloids is assessment of cross-seeding efficiencies between αSyn molecules with familial-PD-associated mutations, e.g., between A30P and A53T [53]. Evaluation of aggregation formation and cross-reactions of in vitro-translated GFP-fusion mutant αSyn demonstrated that αSyn mutants with A53T, H50Q, or E46K spontaneously form large aggregates and efficiently co-aggregate with each other, whereas they do not co-aggregate with the wild-type or G51D or A30P mutants. The latter group of three αSyn species formed smaller aggregates and co-aggregate with each other [54]. These findings suggested existence of two mutually exclusive aggregation paths of αSyn amyloid formation. Cross-seeding experiments demonstrated contribution of the regions N-terminal- and C-terminal to the Greek-key moiety to efficient propagation and strain-specific properties of αSyn amyloids, as demonstrated by in-vitro fibril formation and in vivo inoculation experiments with various human-mouse chimeric αSyn [55], or with N- and C-terminally truncated mutant αSyn [56]. Not only affecting cross-seeding efficiencies between αSyn molecules, strain-specific structures of αSyn amyloids can also affect interactions with other proteins. For instance, induction of Tau pathologies by αSyn preformed fibrils is a strain-specific phenomenon. Two in vitro-formed strains of αSyn amyloids with distinct proteolytic-fragment patterns exhibited different efficiencies in cross-seeding Tau and induction of phosphorylated Tau in vivo. The results demonstrated that the N-terminal region was important for the cross-seeding of Tau [57]. Whether cross-seeding of other proteins by αSyn amyloids, e.g., Aβ [58] or amylin [59], is also strain-dependent warrants further investigation.

**Figure 2:**
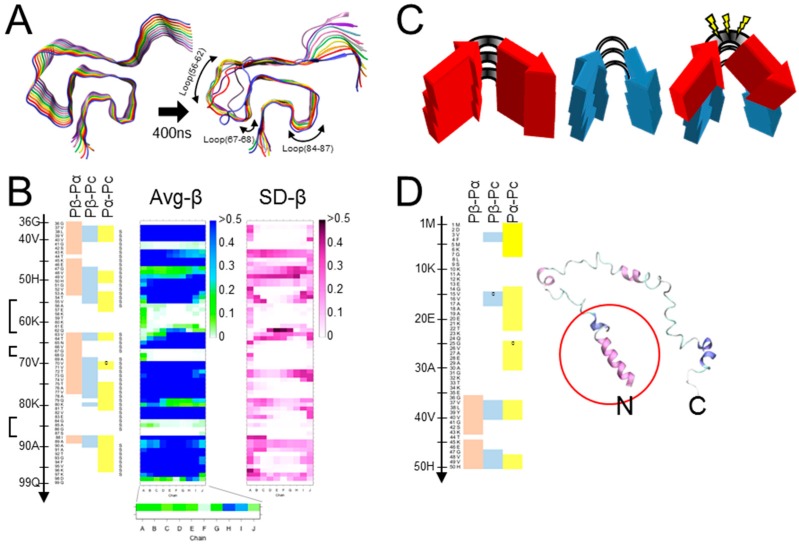
Correlation between results of MD simulation and secondary-structure prediction. (**A**). The “Greek-key” conformation of αSyn amyloid (PDB ID: 2n0a) (left). The status of the stack of αSyn amyloid after 400 ns of MD simulation (right). The arrows indicate the three loops by or in which Ile-substitution mutations are introduced. Protocols for the MD simulations are described in [61] and in the Supplementary Protocols. The ten αSyn molecules in the stack are designated as chain A (blue, the nearest) to J (gray) (Figure S1A). (**B**). (Left) Propensity profiles predicted from the primary structure of αSyn by a neural network secondary structure prediction [62]. The heatmap exhibits the magnitude correlations between the conventional β-sheet propensity (Pβ), α-helix propensity (Pα) and coil propensity (Pc) by the new set of parameters, (Pβ-Pα), (Pβ-Pc) and (Pα-Pc), where zero- (“0”) and positive-value residues of each are indicated in red, blue and yellow, respectively. The three loops in Figure 2A are indicated with brackets. (Middle) A heatmap of the average β-sheet propensity (Avg-β) of αSyn amyloid observed in five independent runs of MD simulation. The color codes indicates the proportion of time when a given residue is in β-sheet structures during the runs. For all the heatmaps of Avg-β and SD-β, the vertical and horizontal axes represent the residues 36-99 and the ten chains A-J of the amyloid stack, respectively. (Right) A heatmap of the standard deviation values of β-sheet propensities (SD-β) of the αSyn amyloid observed in the MD simulation of five independent runs. (**C**). A schematic illustrating a hypothetical local incompatibility between the template amyloid and a heterologous substrate in a cross-seeding reaction. The red peptide has a non-flexible loop, whereas the blue peptide has a more flexible loop. When the red peptide is cross-seeded by the blue amyloid, the substrate red peptide might undergo strain from the incompatibility with the actual structure. (**D**). (Left) A predicted propensity profiles of the N-terminal region of αSyn encompassing the residues 1-50. (Right) A final snap shot of native-form αSyn (PDB ID: 2kkw) after 5 ns of MD simulation without any micelle: Only the N-terminal side encompassing 1-100 was used. Note that regions with high (Pα-Pc) values relatively maintain α-helix structure (red circle). N and C, N- and C-terminus, respectively.

Atomic-level structures of in vitro-formed αSyn amyloids were determined either by ssNMR or cryo-EM [17,23,24]. The conformation determined by ssNMR exhibited a “Greek-key” conformation encompassing residues 35 to 99 with intricate interactions among the constituent β-sheets [17]. Although the majority of αSyn fibrils observed in PD brains were intertwined two-protofibril forms [60], the Greek-key αSyn amyloid was rather stable in MD simulations of 400 ns, even without another protofibril (Figure 2A,B, middle and right panels) [61]. Regardless of relative instability in the region 47–61 of the stack-end molecules, the overall stability of the amyloid stack was sufficient to evaluate the effects of various mutations, e.g., G51D or A53T, on the amyloid structures [61]. The detailed structures of αSyn amyloids determined by cryo-EM revealed co-existence of two apparently distinct types of fibrils formed under the same conditions. Both the polymorphs, “rod” and “twister”, consisted of two intertwined protofibrils and shared the common amyloid “kernel,” but they had different inter-protofibrillar interfaces [24]. The authors therefore postulated that the inter-protofibrillar interface might be an important determinant of αSyn amyloid strains. Although the rod type and the one reported by Guerrero-Ferreira et al. (PDB ID: 6h6b) had Greek-key conformations, they were slightly different from the one identified with ssNMR (PDB ID: 2n0a) in that the side chain of Ala53 pointed outward to constitute the inter-protofibril interface and that the highly-charged region 56–60 formed a β-sheet. Whether the two polymorphs represent two different strains, rather than structural polymorphs of the same strain with the same kernel, would need to be corroborated by strain-specific biological properties of each polymorph.

## 4. Insights from MD Simulations of αSyn Amyloids

The detailed structures of Tau and αSyn amyloids suggested strain diversification by alterations in positions of β-arches, pairing patterns of cross-β spines, and inter-protofibrillar interfaces, which greatly depend on intricate interactions among the constituent β-sheets. On the other hand, the incompatibility between Pick’s Tau amyloid and 4R-Tau substrate by steric conflicts due to the branched Cβ of Val_300_ demonstrated that strain barriers can be also posed by such local factors. We hypothesized that conflicts between the local structures of the template amyloid and the corresponding local intrinsic propensity of the substrate peptides can cause a strain barrier, and posited that the intrinsic propensities are predictable by a neural-network secondary structure prediction algorithm (http://cib.cf.ocha.ac.jp/bitool/MIX/) [62,63]. Although the algorithm was originally designed for monomeric proteins, we thought the singular structural feature of in-register parallel β-sheet amyloids would allow for application (Figure 1A). By comparing predicted propensities, i.e., β-sheet propensity (Pβ), α-helix propensity (Pα), and coil propensity (Pc), of PrPs from various species, we previously hypothesized that compatibility of β-arches between PrP^Sc^ and PrP^C^ contributes to species barriers (Figure 2C) [63]. Here we test the hypothesis of compatibility between the local conformations and the propensity of substrates with MD simulation, which enable observation of the influences exclusively of the conformations on behaviors of the peptides without any interference from other factors or proteins.

**Figure 3:**
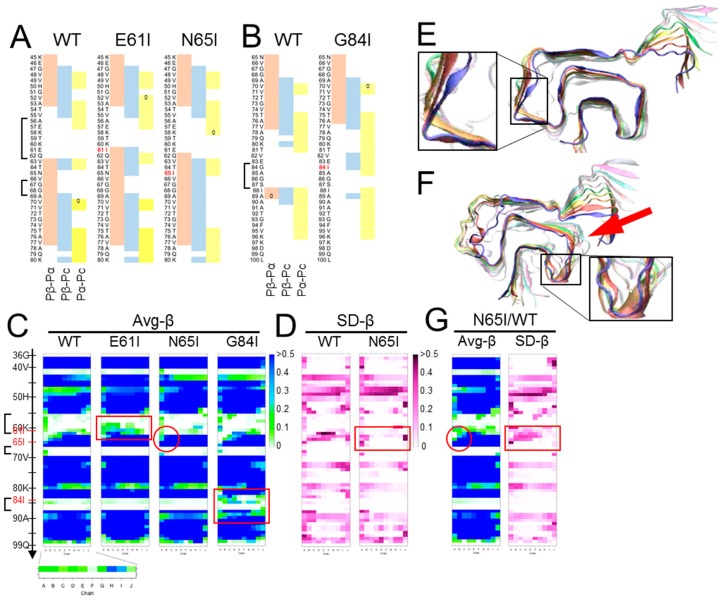
Inappropriately high β-sheet propensity in a loop region can destabilize amyloid structures. (**A**) and (**B**). Comparison of predicted propensity profiles between WT-, E61I-, N65I- and G84I-αSyn. The mutation sites are indicated in red letters. Isoleucine substitutions were introduced near the loop regions based on the assumption that loops with higher β-sheet propensities are less flexible. The brackets indicate the three loops in Figure 2A. (**C**). Comparison of heatmaps of Avg-β between WT-, E61I-, N65I- and G84I-αSyn. They represent five (for WT) or three (for the others) independent simulations. Note that all the mutants have higher Avg-β values near the mutations (more green/blue cells in the red boxes for E61I and G84I, and in the red circle for N65I). (**D**). Comparison of the heatmaps of SD-β between WT- and N65I-αSyn. They represent five and three independent runs, respectively. Note that the region comprising N65I mutation shows more stable β-sheet with smaller SD than WT-αSyn (red box). (**E**). A final snap shot of E61I-αSyn after 400 ns of simulation. The mutation tended to stabilize the global structures of the amyloid and occasionally induced new β-sheets in the loop (56–62) (inset). (**F**). A final snap shot of G84I-αSyn after 400 ns of simulation. The mutation destabilized structures around the mutation and the adjacent regions (arrow). Occasionally β-strands were temporarily induced in the loop (inset). The differential effects of the isoleucine substitutions on the amyloid structures might be attributable to the shapes of the loops. The loop (56–62) is long and makes obtuse angles with the flanking β-strands. The loop (67–68) is short but rather flexible with two glycine residues in a row. The loop (84–87) is a U-shaped loop with a relatively-small turning radius, which could not accommodate Cβ-branching side chain of Ile. (**G**). Heatmaps of a hetero-oligomer amyloid containing N65I-αSyn in chain A-E and WT-αSyn in chain F-J (N65I/WT). Despite N65I mutation, Avg-β and SD-β values of chain A-F are similar to those of WT (red circle and red box).

We chose the “Greek-key” αSyn amyloid (PDB ID: 2n0a) [17] as a representative model of an in-register parallel β-sheet amyloid, because of its intricately-interacting β-sheets, absence of proline, and paucity of aromatic amino acids, wherein its π–π interactions are not fully represented by the used force field. First, we compared predicted propensities with the results of MD simulations of the wild-type (WT) αSyn amyloid [61] to assess the relationship of the predicted propensities and the actual structures of αSyn amyloids in silico. Here, we used a set of modified parameters, Pβ-Pα, Pβ-Pc, and Pα-Pc, to clearly visualize magnitude correlations between two of the three conventional parameters, while the algorithm originally assigns a secondary structure of the highest value to a given residue, to assess whether the magnitude correlations between two have any significance on the structures. These predicted propensities exhibited some correlation with the actual average β-sheet propensity from the MD simulations (Figure 2B), suggesting certain influences of intrinsic propensities on structures of the amyloid. Positive (Pα-Pc) values (Figure 2B, left panel) indicated high α-helix propensities in theory, and indeed the N-terminal region with positive (Pα-Pc) values maintained a helix in the MD simulation of a native αSyn (PDB ID: 2kkw [64]), even in the absence of micelles (Figure 2D). The β-sheet in the region 89-95 with positive-(Pα-Pc) values (Figure 2B) yielded a similar contribution of high α-helix propensities to amyloid formation as reported in the literature, which explains why amyloidogenic proteins often have α-helices in native conformations, e.g., Aβ and PrP [65,66,67,68]. Given the correlation of the predicted propensity profiles with the structures of αSyn amyloid in silico, next, we introduced substitution mutations of isoleucine around the three loops encompassing residues 56–62 [loop(56–62)], 67–68 [loop(67–68)], and 84–87 [loop(84–87)] (Figure 2A), specifically Glu61Ile (E61l), Asn65Ile (N65I), and Gly84Ile (G84I), respectively (Figure 3A,B). Then, we analyzed their predicted propensities. G61I locally raised (Pβ-Pα) and (Pβ-Pc) values (Figure 3A, middle). N65I raised (Pβ-Pc) values in the loop (67–68) and the adjacent β-strands (Figure 3A, right). G84I raised (Pα-Pc) values to positivity through the loop and changed the positive-(Pβ-Pc) spot at the residue 80 to the wider one encompassing 82–84 (Figure 3B).

MD simulations of homo-oligomers of mutant αSyn were performed with the same conditions as outlined in [61] and briefly described in the Supplementary Protocol and Figure S1. Influences of N65I were subtle but the β-sheet encompassing 62–66 was more stabilized particularly on the chain-A side (Figure 3C, red circle) with smaller SD-β values than those of WT (Figure 3D). E61I occasionally induced β-strands in the loop (56–62) and tended to stabilize the amyloid stack (Figure 3E, inset). In contrast, G84I substantially destabilized the loop (84–87) and the adjacent structures (Figure 3F, arrow). In accordance with the raised local β-sheet propensity, β-strands were temporarily induced in loop (84–87) (Figure 3F, inset) but could not form a stable β-sheet. Those varied effects of isoleucine substitutions suggested that raised β-sheet propensity either stabilizes or destabilizes the loops depending on their shapes, lengths, flexibility, and orientation of the side chain; the side chain of N65I pointed outward of the β-arch, while those of E61I and G84I inward. A “U-shaped” loop with small turning radius like loop (84–87) might be destabilized because the space inside the β-arch is too small to accommodate the side chain of Ile with Cβ branching inside. This provides a proof-of-concept to the hypothesis that incompatibility between the intrinsic propensities of the substrate with the actual local structures of the template amyloid affects stability of the amyloid [63]. To note, the amyloids contained only mutant αSyn without any mismatch in the primary structures among the peptides in the stack. In seeding reactions of amyloids or PrP^Sc^ prions, homology in the primary structures between the seed and the substrate is an important determinant of seeding efficiency; however, the present result corroborated that compatibility of intrinsic propensity of the substrate with local structures of the amyloid is more important than the sequence homology. This helps to interpret the singular inter-species transmissions of PrP^Sc^ prions wherein even secondary transmissions are inefficient, despite the homology between the PrP^Sc^ in the inoculum and the host PrP [69,70]. Interestingly, the stabilizing effects of E61I or N65I in the homo-oligomers were affected in hetero-oligomers by the combined WT αSyn (Figure 3G). As peptides in an amyloid are incessantly moving in a fine vibratory manner with a twisting tendency, the hetero-oligomers might have discordance in the motions between the heterologous peptides that eventually lead to destabilization.

## 5. Significance of Stack-End Molecular Behavior

MD simulations of various mutant αSyn amyloids demonstrated that behaviors of the stack-end molecules, i.e., which β-sheets of the stack-ends are stable, were highly varied depending on the primary structures, despite being in the same conformation [61]. Whether the behaviors of the stack-end molecules (Figure 2B, chain A or J) affect incorporation of the substrate to the stack to pose a strain barrier is yet to be investigated; however, if they do, they could modulate levels of barriers without substantial conformational changes depending on environment, e.g., pH or salt strength. Indeed, seeding reactions of amyloids are often affected by reaction conditions [49]. This may also explain why different strains have apparently similar secondary structures [71,72,73].

## 6. Implications about PrP^Sc^ Prion

We previously investigated inter-species transmissions of mammalian prions with a focus on PrP^Sc^ as an in-register parallel β-sheet amyloid [63]. Indeed, in-register parallel β-sheet amyloids show many similar properties to PrP^Sc^. For example, the existence of different proteolytic-fragment patterns of Tau and αSyn amyloids [31,49,52] are reminiscent of the 19-kDa and 21-kDa fragments of PrP^Sc^ prions [74]. Besides, those polymorphs of Tau and αSyn amyloids are associated with distinct clinicopathological features [14,52]. These proteins are good surrogate models for PrP^Sc^ and may elucidate mechanisms of strain diversification and translation of strain-specific structures, particularly because of the aforementioned advantages in experiments.

**Figure 4:**
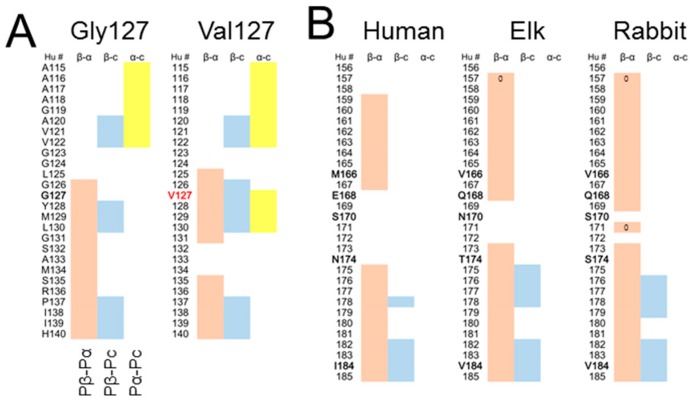
Influences of primary structures on predicted propensity profiles of PrP. (**A**). Comparison of predicted propensity profiles between two codon-127 polymorphs of human PrP, Gly127 (left) and Val127 (right). (**B**). Comparison of predicted propensity profiles of H1~H2 regions between human, elk and rabbit PrPs. Hu #, residue numbers in human numbering. Species-specific residues are denoted in bold letters.

MD simulations of the mutant αSyn demonstrated that inappropriately high intrinsic β-sheet propensity of loops in certain shapes, e.g., a U-shape, can destabilize the local structures of the amyloid. Inversely, local structures of a given amyloid might be inferable from mutations around putative loop regions; if a mutation that raises local β-sheet propensity inhibits incorporation of the mutant peptide into the amyloid, the local structure around the mutation could be a U-shaped loop which cannot accommodate newly-induced β-sheets or the bulky side chain with Cβ branching. Interestingly, the human PrP with an anti-prion polymorphism Val127 [75] shows positive (Pβ-Pc) values at the residues 126–127 where the PrP with Gly127 has negative values (Figure 4A). As residue 127 is located at the end of the flexible region intervening two high-(Pβ-Pc) regions, AVVGGLGG_127_YMLGS, like the residue 84 of the αSyn amyloid, it may also affect structures of the loop and destabilize the surrounding structures. Moreover, as other residues associated with strain barriers are often located near presumably flexible regions, e.g., codon 129 of human PrP [74] and codon 136 of ovine PrP [76], the similar mechanism might affect the heterologous transmission efficiencies as well. Notably, in the Groveman’s in-register parallel β-sheet model of PrP^Sc^, i.e., PIRIB model, residue 129 is located in the middle of a long straight stretch [8]. If minimal surface area of a protofibril of amyloid is thermodynamically favorable as in a micelle, curling like a Greek-key conformation is advantageous in this regard. Given the relative instability in the N-terminal region of the PIRIB model [8,42], an in-register parallel β-sheet model of PrP^Sc^ with a β-arch around residue 129 might be worth considering. On the other hand, the Silva’s four-rung β-solenoid model of PrP^Sc^ has a β-arch centering the Leu_125_ [77]. Although theoretically its structural stability can be similarly affected by Val127 as in the in-register parallel amyloid model, low sequence homology between layers of β-arch of the β-solenoid model may modify the effects, just as influences of a mutation were masked in heterologous β-arches in the hetero-oligomer αSyn amyloid (Figure 3G). Therefore, investigations specifically into β-solenoids for evaluation of influences of mutations are necessary to test this view.

The region between the first and second α-helices (H1~H2) can be one of the main interfaces between substrate PrP^C^ and template PrP^Sc^, i.e., their first interaction site [78]. Although the predicted β-sheet propensities of this region are not necessarily high because of coil propensity (Figure 4B, left), the conformation of native PrP^C^ possibly restricts the mobility of H1~H2 region to make it more prone to β-sheet formation in effect than the predicted propensity. Remarkably, predicted β-sheet propensities of H1~H2 are highly varied among species (Figure 4B), and the mismatches in the propensities in H1~H2 may underlie species barriers by hampering stable β-sheet formation between the PrP^Sc^ and PrP^C^ monomer; if the interface cannot convert to stable β-sheet structures, the conversion reaction would not spread to the rest of the molecule. Other regions with high β-sheet propensities, e.g., second or third α-helix, are stably structured in the native PrP^C^, and unsuitable for interfaces because they demand the energy to unfold before interaction with the template PrP^Sc^. There can be another interface in more N-terminal regions. Considering that Pro102Leu substitution of Gerstman-Sträussler-Scheinker syndrome (GSS) raises local β-sheet propensities in the N-terminal-side region, the 7-kDa protease-resistant fragment of GSS derived from the N-terminal region [79] may reflect preferential interactions of the region with PrP^Sc^ as a consequence of the raised local propensity. Interestingly, Fukuoka-1 mouse-adapted prion, which was originally derived from GSS, has distinct structures in H1~H2 from that of other mouse-adapted strains RML or 22L [80]. If pairing patterns of cross-β spines depend on positions of the initiating amyloid core as hypothesized above, the differences might be explainable: β-sheets of H1~H2 form cross-β spines with the C-terminal region of PrP in the latter strains, while they form cross-β spines with another region in PrP^Sc^ of GSS.

## 7. Implications and Conclusions

After strain-specific structures of amyloids are unveiled, the next question to be addressed is how those structures are translated into strain-specific clinicopathological features. The induction of conformational changes by pathogenic amyloids may also occur to non-amyloidogenic proteins which have optimal intrinsic propensities, via cross-seeding reactions between non-amyloidogenic protein and amyloids. If the same principles as that for between amyloidogenic proteins operate, non-amyloidogenic proteins which preferentially interact with a given amyloid might be predictable from the primary structure.

Herein, we reviewed recent advances in the structures and mechanisms of strain diversity of in-register parallel β-sheet amyloids, particularly Tau and αSyn, and discussed implications regarding PrP^Sc^ prions. Strain diversification of in-register parallel β-sheet amyloids seems to have multiple mechanisms due to intricate interactions between the β-sheets and local structures. As in-silico methodology proves to be useful presumably because of the singular structural features, more proactive use of it would foster further advances in our knowledge.

## Supplementary Materials

The following are available online at http://www.mdpi.com/1999-4915/11/2/110/s1, Figure S1: Supplementary information about the MD simulation., Table S1: Raw data for the heatmaps, Supplementary Protocols: Protocols for the MD simulation.
