# Supplementary material for: Mechanisms of Strain Diversity of Disease-Associated in-Register Parallel β-Sheet Amyloids and Implications About Prion Strains
Source: Viruses. 2019 Jan 28;11(2):110. doi: 10.3390/v11020110 (PMC6410106; doi:10.3390/v11020110)
Supplement: Supplementary file 1 [file viruses-11-00110-s001.zip › Supplementary Materials/Supplementary Protocols & Figure S1.docx]

**Supplementary Protocols**

**Modelling Structure**

We used the Greek-key conformation of the αSyn amyloid (PDB ID: 2n0a) as the starting structure [1]. Because our interest was in the general properties of in-register parallel β-sheet amyloids rather than αSyn itself, we removed the N- and C-terminal disordered regions (residues 1–35 and 100–140) to reduce calculation burdens. The N- and C-termini were acetylated and methylated using AmberTools16 [2], respectively, to minimize electrostatic interactions at these termini. For simplicity of description, we refer to each layer from top to bottom as chain A to J (**Supplementary Fig S1A, right panel**). For modelling αSyn mutants, we used SCWRL4 [3]. Modelled amyloids were solvated with a rhombic dodecahedron water box with a minimum wall distance of 12 Å using GROMACS (version 5.0.4). Na^+^ and Cl^–^ ions were randomly placed to neutralize the system and yield a net concentration of 150 mM NaCl. The protonation state of the histidine at the residue 50 was fixed as the N^δ^-H tautomer (HID form in AMBER naming convention) in all simulations.

**MD simulations**

GROMACS (versions 5.0.4 and 5.1.2) [4] with the AMBER ff99SB-ILDN force field [5] was used for MD simulations of the αSyn amyloids with the TIP3P water model [6]. The system was initially minimized for 5,000 steps with the steepest descent method, followed by 2,000 steps with the conjugate gradient method. During minimization, heavy atoms of the amyloids were restrained with a harmonic potential with a force constant of 10.0 kcal/mol•Å^2^. After minimization, the temperature of the system was increased from 0 to 310 K during a 1 ns simulation with the restraints. Next, a 1 ns equilibration run was performed by gradually reducing the restraints from 10.0 kcal/mol•Å^2^ to zero and subsequent equilibration was performed in the NPT ensemble for 2 ns at 310 K and 1 bar. Production runs were carried out for 400 ns in the NPT ensemble at 310 K and 1 bar (**Supplementary Fig S1B**). We used the velocity-rescaling scheme [7] and the Berendsen scheme [8] for thermostat and barostat, respectively. The LINCS algorithm [9] was used to constrain all bonds with hydrogen atoms, allowing the use of a 2 fs time step. Electrostatic interactions were calculated with the Particle-mesh Ewald method [10]. The cut-off length was set to 12 Å for Coulomb and van der Waals interactions. The Verlet cut-off scheme [11] was used for neighbour searching. Five MD simulations were carried out for the wild-type, and three MD simulations performed for the other mutants. Trajectory snapshots were saved every 10 ps for the MD simulations.

**Analyses**

The first 100 ns of each MD trajectory was discarded, except for calculation of the root-mean-square deviation (RMSD). Thus, 30,001 snapshots (trajectory from 100 to 400 ns) were used for the analysis. The RMSD for backbone atoms of every chain was calculated using the *gmx rms* program in GROMACS package. The ssNMR structure was used as the reference structure. The secondary structure content during the simulations was calculated using DSSP [12,13] and *gmx do_dssp* in GROMACS. Hydrophobic contacts were analysed using *PyInteraph* [14]. All molecular structures for αSyn amyloids were drawn by UCSF Chimera (version 1.12) [15].

**References**

1. Tuttle, M.D.; Comellas, G.; Nieuwkoop, A.J.; Covell, D.J.; Berthold, D.A.; Kloepper, K.D.; Courtney, J.M.; Kim, J.K.; Barclay, A.M.; Kendall, A.; Wan, W.; Stubbs, G.; Schwieters, C.D.; Lee, V.M.Y.; George, J.M.; Rienstra, C.M. Solid-state NMR structure of a pathogenic fibril of full-length human α-synuclein. *Nat. Struct. Mol. Biol.* **2016**, *23*, 409–415, doi:10.1038/nsmb.3194.

2. Case, D.A.; Betz, R.M.; Cerutti, D.S.; Cheatham, III, T.E.; Darden, T.A.; Duke, R.E.; Giese, T.J.; Gohlke, H.; Goetz, A.W.; Homeyer, N.; Izadi, S.; Janowski, P.; Kaus, J.; Kovalenko, A.; Lee, T.S.; LeGrand, S.; Li, P.; Lin, C.; Luchko, T.; Luo, R.; Madej, B.; Mermelstein, D.; Merz, K.M.; Monard, G.; Nguyen, H.; Nguyen, H.T.; Omelyan, I.; Onufriev, A.; Roe, D.R.; Roitberg, A.; Sagui, C.; Simmerling, C.L.; Botello-Smith, W.M.; Swails, J.; Walker, R.C.; Wang, J.; Wolf, R.M.; Wu, X.; Xiao, L.; Kollman, P.A. *AmberTools16. University of California, San Francisco.*; 2016;

3. Krivov, G.G.; Shapovalov, M. V; Dunbrack, R.L. Improved prediction of protein side-chain conformations with SCWRL4. *Proteins Struct. Funct. Bioinforma.* **2009**, *77*, 778–795, doi:10.1002/prot.22488.

4. Abraham, M.J.; Murtola, T.; Schulz, R.; Páll, S.; Smith, J.C.; Hess, B.; Lindahl, E. GROMACS: High performance molecular simulations through multi-level parallelism from laptops to supercomputers. *SoftwareX* **2015**, *1*–*2*, 19–25, doi:https://doi.org/10.1016/j.softx.2015.06.001.

5. Lindorff-Larsen, K.; Piana, S.; Palmo, K.; Maragakis, P.; Klepeis, J.L.; Dror, R.O.; Shaw, D.E. Improved side-chain torsion potentials for the Amber ff99SB protein force field. *Proteins Struct. Funct. Bioinforma.* **2010**, *78*, 1950–1958, doi:10.1002/prot.22711.

6. Jorgensen, W.L.; Chandrasekhar, J.; Madura, J.D.; Impey, R.W.; Klein, M.L. Comparison of simple potential functions for simulating liquid water. *J. Chem. Phys.* **1983**, *79*, 926–935, doi:10.1063/1.445869.

7. Bussi, G.; Donadio, D.; Parrinello, M. Canonical sampling through velocity rescaling. *J. Chem. Phys.* **2007**, *126*, 14101, doi:10.1063/1.2408420.

8. Berendsen, H.J.C.; Postma, J.P.M.; van Gunsteren, W.F.; DiNola, A.; Haak, J.R. Molecular dynamics with coupling to an external bath. *J. Chem. Phys.* **1984**, *81*, 3684–3690, doi:10.1063/1.448118.

9. Hess, B. P-LINCS:  A Parallel Linear Constraint Solver for Molecular Simulation. *J. Chem. Theory Comput.* **2008**, *4*, 116–122, doi:10.1021/ct700200b.

10. Essmann, U.; Perera, L.; Berkowitz, M.L.; Darden, T.; Lee, H.; Pedersen, L.G. A smooth particle mesh Ewald method. *J. Chem. Phys.* **1995**, *103*, 8577–8593, doi:10.1063/1.470117.

11. Páll, S.; Hess, B. A flexible algorithm for calculating pair interactions on SIMD architectures. *Comput. Phys. Commun.* **2013**, *184*, 2641–2650, doi:https://doi.org/10.1016/j.cpc.2013.06.003.

12. Touw, W.G.; Baakman, C.; Black, J.; te Beek, T.A.H.; Krieger, E.; Joosten, R.P.; Vriend, G. A series of PDB-related databanks for everyday needs. *Nucleic Acids Res.* **2015**, *43*, D364–D368, doi:10.1093/nar/gku1028.

13. Kabsch, W.; Sander, C. Dictionary of protein secondary structure: Pattern recognition of hydrogen-bonded and geometrical features. *Biopolymers* **1983**, *22*, 2577–2637, doi:10.1002/bip.360221211.

14. Tiberti, M.; Invernizzi, G.; Lambrughi, M.; Inbar, Y.; Schreiber, G.; Papaleo, E. PyInteraph: A Framework for the Analysis of Interaction Networks in Structural Ensembles of Proteins. *J. Chem. Inf. Model.* **2014**, *54*, 1537–1551, doi:10.1021/ci400639r.

15. Pettersen, E.F.; Goddard, T.D.; Huang, C.C.; Couch, G.S.; Greenblatt, D.M.; Meng, E.C.; Ferrin, T.E. UCSF Chimera—A visualization system for exploratory research and analysis. *J. Comput. Chem.* **2004**, *25*, 1605–1612, doi:10.1002/jcc.20084.

**Supplementary Figure S1**

**
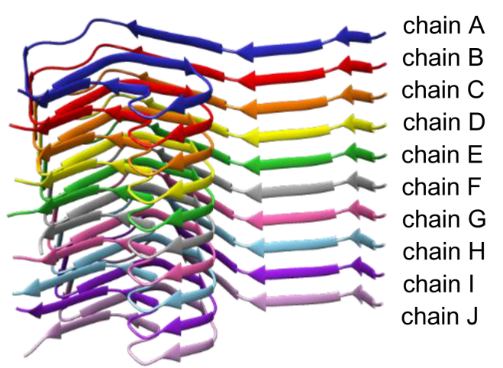
A**


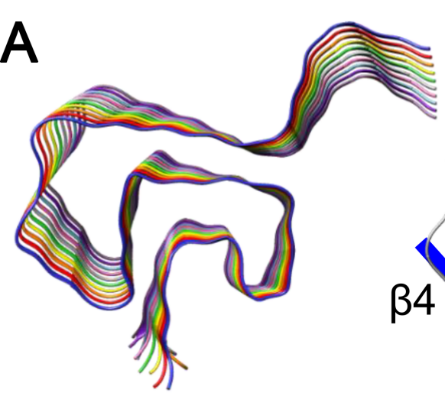


**
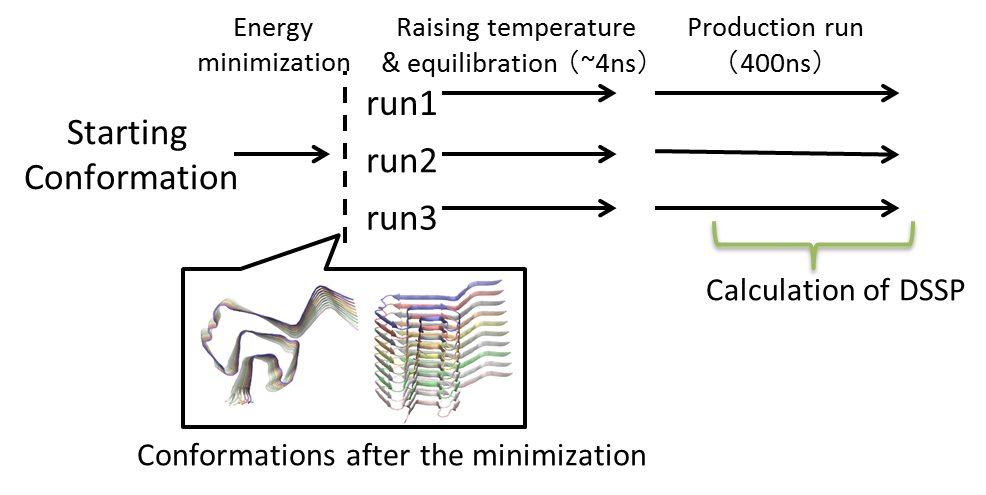
B**

**Figure S1. Supplementary information of the MD simulation of αSyn amyloid.**

**A.** A schematic illustration of the ten-layer Greek-key αSyn amyloid (PDB ID: 2n0a). The layers were designated as chain A to chain J (right panel).

**B.** A schematic illustration overviewing the time course of MD simulation procedure.
